# Tau passive immunization blocks seeding and spread of Alzheimer hyperphosphorylated Tau-induced pathology in 3 × Tg-AD mice

**DOI:** 10.1186/s13195-018-0341-7

**Published:** 2018-01-31

**Authors:** Chun-ling Dai, Wen Hu, Yunn Chyn Tung, Fei Liu, Cheng-Xin Gong, Khalid Iqbal

**Affiliations:** Department of Neurochemistry, Inge Grundke-Iqbal Research Floor, New York State Institute for Basic Research in Developmental Disabilities, 1050 Forest Hill Road, Staten Island, NY 10314 USA

**Keywords:** Tauopathy, Alzheimer’s disease, Immunotherapy, Tau transmission, Amyloid plaques

## Abstract

**Background:**

Accumulating evidence indicates that Tau pathology can spread from neuron to neuron by intake and coaggregation of the hyperphosphorylated Tau (p-Tau) seeds with the host neuron protein. Thus, clearance of Tau seeds by immunization with Tau antibodies could provide a potential therapeutic opportunity to block the spread of the pathology in Alzheimer’s disease (AD) and other tauopathies. We report prevention of the seeding and spread of tau pathology with mouse monoclonal antibody 43D against the N-terminal projection domain of Tau (Tau 6–18) in triple-transgenic AD (3 × Tg-AD) mice.

**Methods:**

Female 11- to 12-month-old 3 × Tg-AD mice were intravenously immunized weekly for 6 weeks with 15 μg/injection of mouse monoclonal antibody 43D or with mouse immunoglobulin G as a control. AD p-Tau isolated from a frozen autopsied AD brain was unilaterally injected into the right hippocampus on the day of the second dose of immunization. Tau pathology and its effect on Aβ pathology were assessed by immunohistochemical staining.

**Results:**

We found that the injection of AD p-Tau into the hippocampus of 11- to 12-month-old 3 × Tg-AD mice time-dependently induced Tau aggregation in the hippocampus and promoted the spread of Tau pathology to the contralateral hippocampus. Tau pathology was observed as early as 6 weeks after AD p-Tau injection. Tau pathology templated by AD p-Tau was thioflavin-S-positive and was about two-fold greater than that seen in naive 18-month-old 3 × Tg-AD mice; Tau pathology in the latter was thioflavin-S-negative. Immunization with Tau antibody 43D dramatically blocked AD p-Tau seeding in the ipsilateral hippocampus and inhibited its propagation to the contralateral side in 3 × Tg-AD mice. Furthermore, AD p-Tau injection enhanced the amyloid plaque load in the ipsilateral side, and immunization with 43D showed a tendency to attenuate it.

**Conclusions:**

These findings indicate that AD p-Tau-injected 3 × Tg-AD mice represent a practical model to study the seeding and spread of Tau pathology, their effect on Aβ pathology, and the effect of Tau immunotherapy on both Tau and Aβ pathologies. Immunization with Tau antibody 43D to Tau 6–18 can prevent the seeding and spread of Tau pathology, making it a potential therapeutic treatment for AD and related tauopathies.

## Background

Alzheimer’s disease (AD), characterized by progressive loss of memory and other cognitive functions, is the most common cause of dementia. The two major histopathological hallmarks in the AD brain are extracellular senile plaques, consisting of amyloid-β (Aβ) peptides [1], and intracellular neurofibrillary tangles (NFTs), composed of abnormally hyperphosphorylated Tau (AD p-Tau) protein [2]. Tau, a microtubule-associated protein, normally stabilizes neuronal microtubules and promotes their assembly. However, in certain pathological conditions, hyperphosphorylated Tau causes the formation of insoluble aggregates that are toxic to neurons [3–6]. The Tau pathology made up of the p-Tau is also a hallmark of several neurodegenerative disorders known as *tauopathies*, which include frontotemporal dementia, corticobasal degeneration, progressive supranuclear palsy, Pick disease, Guam Parkinsonism-dementia complex, and chronic traumatic encephalopathy. Currently, there are no effective treatments available for AD and related tauopathies.

Studies using autopsied brains indicate that neurofibrillary pathology in the brains of patients with AD starts in the entorhinal/perirhinal cortex and spreads anatomically in a defined pattern to the limbic system and eventually the isocortex [7, 8]. Only recently, the stereotypical pattern of Tau pathology progression similar to that in AD was shown experimentally in mice. Injection of brain extract from P301S mutated Tau-expressing mice harboring NFTs or brain homogenates from human tauopathies into the brain of transgenic wild-type human Tau (h-Tau)-expressing mice induced the assembly of wild-type h-Tau into filaments and the spread of pathology from the site of injection to neighboring brain regions [9, 10]. Intracerebral inoculation of young PS19 mice overexpressing P301S mutated h-Tau with synthetic preformed aggregates assembled from recombinant full-length Tau or truncated Tau containing four microtubule-binding repeats resulted in rapid induction of NFT-like inclusions that propagated from injected sites to connected brain regions in a time-dependent manner [11]. The spread of Tau pathology in brains was further confirmed in the mouse models expressing P301L mutated h-Tau restricted to the entorhinal cortex (EC), in which Tau pathology progressed from EC transgene-expressing neurons to neurons without detectable transgene expression, first to EC neighboring cells, followed by propagation to neurons downstream in the synaptic circuit, such as the dentate gyrus, cornu amonis (CA) fields of the hippocampus, and cingulate cortex [12, 13]. Taken together, these data suggest that a prion-like spread mechanism may drive Tau pathology, leading to synaptic and cognitive deficits in human AD and related tauopathies.

The abnormally hyperphosphorylated/oligomeric Tau released into the extracellular space from the affected neurons is suspected to serve as the seed for the spread of Tau pathology by the ingesting cells [9, 14]. Therefore, immunotherapy against Tau may clear extracellular Tau that is involved in the spread of the pathology and rescue memory deficits. Treatment with antibodies that block the seeding activity in vitro and anti-Tau pS396 and pT231 were reported to inhibit the spread of Tau pathology in Tau-transgenic mice [15, 16].

In a recent study, we found that intrahippocampal injection with AD p-Tau isolated from a frozen autopsied AD brain produced numerous p-Tau tangles and neuropil threads locally and in neocortex lateral to injection and upstream of the hippocampus in h-Tau-transgenic mice [17]. We discovered that immunization with monoclonal antibody 43D against Tau 6–18 reduced both Tau and Aβ pathologies, as well as rescued cognitive impairment in moderate to severe stages of Tau pathology in triple-transgenic AD (3 × Tg-AD) mice [18, 19].

In this article, we report that, in addition to seeding and spread of Tau pathology, AD p-Tau also enhances Aβ plaque load and that immunization with antibody 43D prevents the seeding and propagation of Tau pathology and the promotion of Aβ pathology in 3 × Tg-AD mice. These findings suggest that immunization with 43D can efficiently block both Tau and Aβ pathologies promoted by extracellular Tau seeds. Thus, immunization targeting proximal N-terminal Tau domain Tau 6–18 offers a potential therapeutic opportunity for AD and related tauopathies.

## Methods

### Transgenic mice

The homozygous 3 × Tg-AD mice harboring human APP_SWE_ and Tau_P301L_ transgenes with knock-in PS1_M146V_ under the control of the mouse Thy1.2 promoter, created in the laboratory of Dr. Frank LaFerla [20], were obtained from The Jackson Laboratory (Bar Harbor, ME, USA; https://www.jax.org/strain/004807). Both homozygous male and female 3 × Tg-AD mice with the mixed C7BL/6;129X1/SvJ;129S1/Sv genetic background were bred in the animal colony of the New York State Institute for Basic Research in Developmental Disabilities (Staten Island, NY, USA). Mice had access to food and water ad libitum and were housed (four or five animals per cage) in a pathogen-free facility with 12-h/12-h day/night cycles. The female 3 × Tg-AD mice were reported to develop amyloid plaques starting at ~ 9 months of age and NFTs starting at ~ 12 months of age, respectively, and the pathologies are predominantly restricted to the hippocampus, amygdala, and cerebral cortex [20–22]. Unlike females, male 3 × Tg-AD mice show inconsistent and weak pathology [23–26], and hence only female animals were used in the present study.

**Fig. 1:**
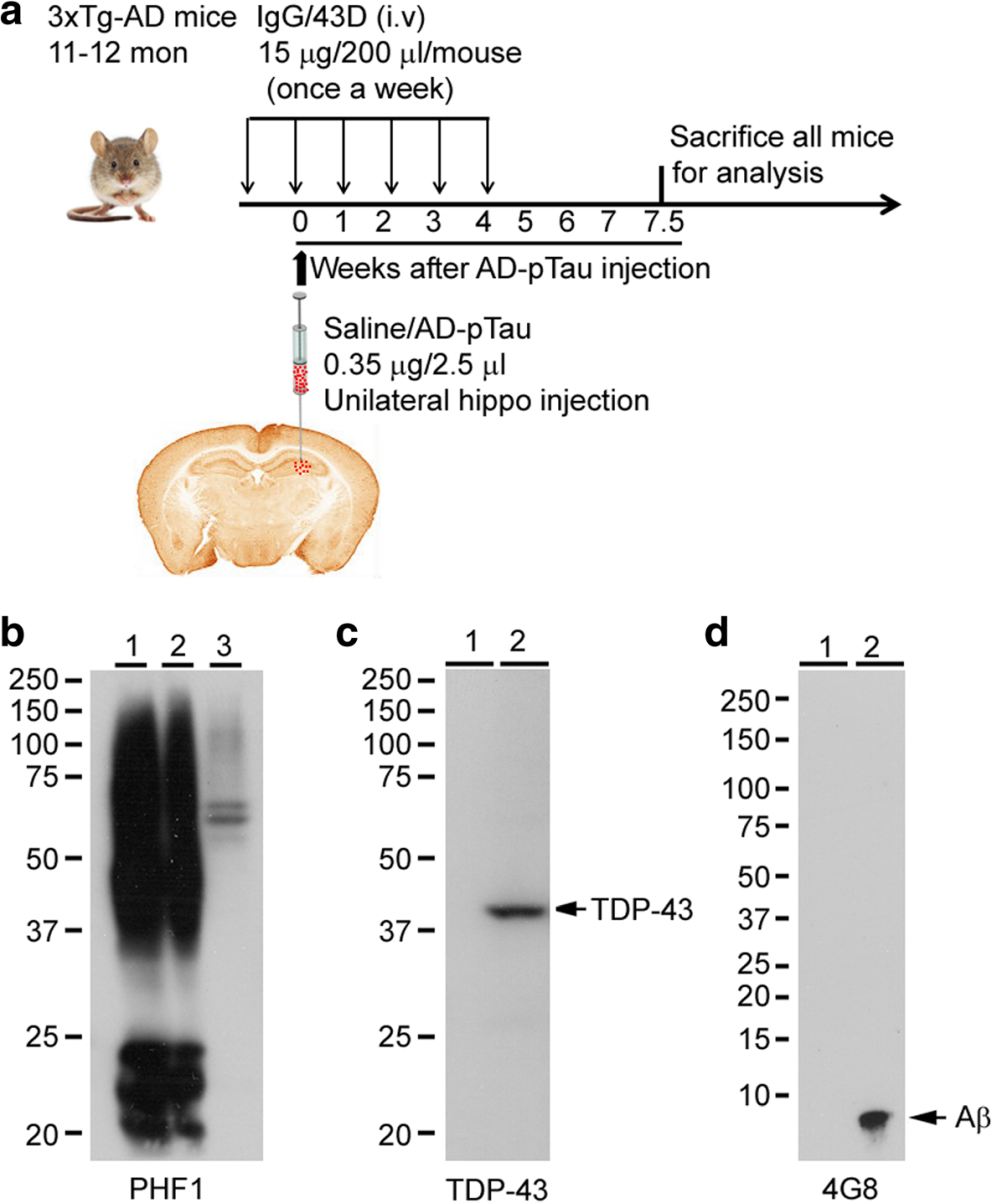
Study design and characterization of Alzheimer’s disease abnormally hyperphosphorylated Tau (AD P-Tau). **a** Female triple-transgenic Alzheimer’s disease (3 × Tg-AD) mice were immunized with 43D or as a control with mouse immunoglobulin G (IgG) (i.v., 15 μg in 200 μl of saline) once weekly for 6 weeks. AD p-Tau (0.35 μg in 2.5 μl of saline) isolated from AD cerebral cortex was unilaterally injected into right hippocampus of 11- to 12-month-old 3 × Tg-AD mice on the day of the second dose of immunization. Saline/43D (*n* = 6), AD p-Tau/43D (*n* = 6), and AD p-Tau/IgG (*n* = 7). **b** Quantities of 5 μg of AD p-Tau (*lane 1*), 2.5 μg of AD p-Tau (*lane 2*), and 7 μg of AD brain extract (15,000 × *g*) were loaded, and blots were developed with PHF-1 antibody. **c** Quantities of 23 μg of AD p-Tau (*lane 1*) and 15 μg of N2a cell lysate as a positive control (*lane 2*) were loaded, and blots were developed with transactive response DNA-binding protein 43 (TDP-43) antibody. **d** Quantities of 23 μg of AD p-Tau (*lane 1*) and 0.1 μg of synthetic amyloid-β (Aβ) (*lane 2*) were loaded, and blots were developed with 4G8 antibody

To minimize the variation, mice used in this study were first grouped by body weight and age, and then the mice from each litter were evenly assigned to different study groups. The grouped mice were randomized into three study groups: (1) AD p-Tau intracerebral injection plus six weekly doses of 43D (AD p-Tau/43D; *n* = 6), (2) AD p-Tau intracerebral injection plus six weekly doses of mouse immunoglobulin G (IgG) (AD p-Tau/IgG; *n* = 7), and (3) saline (vehicle) intracerebral injection plus six weekly doses of 43D (saline/43D; *n* = 6) (Fig. 1a). A separate group of 11- to 12-month-old female 3 × Tg-AD mice that received AD p-Tau injections as above were killed with a 3-week interval to determine the time-dependent AD p-Tau seeding at the injection site (*n* = 2 at each time point). To compare Tau pathology produced by templation with AD p-Tau with that produced by spontaneous development, we employed 18-month-old naive female 3 × Tg-AD mice (*n* = 3).

### AD brain samples

Frozen brain tissue samples from autopsied and histopathologically confirmed AD cases were obtained from the Harvard Brain Tissue Resource Center, McLean Hospital (Belmont, MA, USA).

### Preparation of Tau seeds

Oligomeric, AD p-Tau was isolated from the cerebral cortex of a frozen autopsied AD brain as described by us previously [6, 17, 27]. Briefly, 10% brain homogenate prepared in the buffer (20 mM Tris-HCl, pH 8.0, 0.32 M sucrose, 10 mM β-mercaptoethanol, 5 mM MgSO_4_, 1 mM ethylenediaminetetraacetic acid [EDTA], 10 mM glycerophosphate, 1 mM Na_3_VO_4_, 50 mM NaF) with phosphatase and protease inhibitors was centrifuged at 27,000 × *g* for 30 minutes. The supernatant was further centrifuged at 235,000 × *g* for 45 minutes, and the resulting pellet (AD p-Tau) was collected and washed three times and then resuspended in saline. The AD p-Tau was bath-sonicated using three bursts of 10 seconds each.

### Intracerebral injections of AD p-Tau

As previously reported by us [17], mice were deeply anesthetized with 1.25% tribromoethanol (Avertin; Sigma-Aldrich, St. Louis, MO, USA) and placed in a stereotactic frame. After a craniotomy 1 mm in diameter was made with a motorized minidrill, the Tau seeds were injected using a 10-μl Hamilton syringe custom-made with a 30-gauge/0.5-inch cemented needle (Hamilton Syringe Co., Reno, NV, USA). AD p-Tau was unilaterally injected into the right hippocampus (0.35 μg of Tau in 2.5 μl of saline) of 11- to 12-month-old female 3 × Tg-AD mice. The coordinates were as follows: −2.5 mm anterior/posterior, +2.0 mm medial/lateral to bregma, and −1.8 mm dorsal/ventral to the dura surface. Tau seeds were injected at a rate of 1.25 μl/minute, and the needle was kept in position for 3 minutes before slow withdrawal to prevent leakage of the liquid infused. An identical volume of saline was also injected into the hippocampus of 3 × Tg-AD mice as vehicle controls. The skin was sutured after injection, and the mice were allowed to completely recover on a soft heating pad before they were returned to their home cages.

### Immunizations with Tau antibodies

Female 3 × Tg-AD mice (six to seven mice/group) aged 11 to 12 months old were immunized intravenously through the tail vein with 15 μg of 43D or as a control with mouse IgG in 200 μl of saline once weekly for 6 weeks. One week after the first dose of immunization, mice received an intracerebral injection of AD p-Tau or saline as a vehicle control (Fig. 1a).

### Tissue process

Mice were anesthetized and transcardially perfused with 30 ml of PBS, followed by 20 ml of 4% paraformaldehyde in 0.1 M phosphate buffer. Brains were removed and postfixed in 4% paraformaldehyde in 0.1 M phosphate buffer for 48 h and then processed through 30% sucrose in 0.1 M phosphate buffer until the brain tissue sank to the bottom of the tube. Serial 40-μm coronal brain sections were collected and used in the present study.

### Immunofluoresence and thioflavin-S staining

Free-floating coronal sections were washed in 10 mM PBS (three times, 15 minutes each) and then incubated in 0.3% Triton X-100 for 30 minutes. The sections were again washed in 10 mM PBS (three times, 15 minutes each) and blocked by incubation in blocking solution (5% normal goat serum, 0.1% Triton X-100, and 0.05% Tween 20 in PBS) for 60 minutes. Sections were then incubated with AT8 (phospho-tau at Ser^202^/Thr^205^, 0.2 μg/ml; Thermo Fisher Scientific, Rockfork, IL, USA) or PHF-1 (phospho-tau at Ser^396^/^404^, 1:1000, a gift from Dr. Peter Davies) or both AT8 and rabbit monoclonal antibody against Aβ (D54D2, 1:300; Cell Signaling Technology, Danvers, MA, USA) at 4 °C overnight. On the second day, sections were washed in 10 mM PBS (three times, 15 minutes each), followed by incubation with Alexa Fluor 555-conjugated goat anti-mouse IgG (1:500; Thermo Fisher Scientific), or in case of double immunostaining, in combination with Oregon Green 488-conjugated goat anti-rabbit IgG (1:500; Thermo Fisher Scientific), in 10 mM PBS with 0.05% Tween 20 for 2 h at room temperature. Sections were subsequently washed, mounted onto Superfrost Plus slides (Fisher Scientific, Pittsburgh, PA, USA) and allowed to air-dry for 3 h at room temperature. Sections on the slide were stained with 0.05% thioflavin-S in PBS in the dark for 8 minutes and then washed in 50% ethanol twice for 1 minute each and in water for 3 minutes. Finally, the sections were coverslipped using ProLong Gold Antifade Mounting Medium (Thermo Fisher Scientific). In the study to investigate the time-dependent AD p-Tau seeding in 3 × Tg-AD mice, AT8 antibody and Alexa Fluor 488-conjugated goat antimouse antibodies (1:500; Thermo Fisher Scientific) were used. Images were acquired using a Nikon Eclipse 90i microscope equipped with an EZ C1 laser-scanning confocal microscopy system (Nikon Instruments, Melville, NY, USA). No primary antibody staining was used as a control for all immunohistochemical studies.

### Quantitation of tangles with AT8 and PHF-1 high-intensity staining

The templated Tau pathology by AD p-Tau exhibited a bright immunofluorescence signal as compared with markedly dimmer phospho-Tau staining of nontemplated Tau pathology in the hippocampus. We set high-intensity staining with the gain at 6.40B for × 10 and 6.10B for × 20 objectives in the confocal system, a setting that filtered out the phospho-Tau staining of nontemplated Tau pathology in the hippocampus. In this way, we were able to easily count the number of templated tangles in the mouse brain sections.

Serial whole-brain coronal sections (40 μm) were collected from bregma +0.4 mm to −3.4 mm. The number of tangles in from 22–25 serial coronal sections per mouse (every fourth section of the brain) that stained positive with both thioflavin-S and intensely with AT8 or PHF-1 were counted at × 10 magnification by an experimenter who was blind to this study.

### Immunohistochemical staining

To study the morphology of templated NFT-like pathology, we performed immunohistochemical staining of mouse brain sections with antibodies against different phospho-tau epitopes (i.e., pSer^202^/pThr^205^ [AT8] and pSer^396/404^ [PHF-1]), and we visualized the immunostaining with 3,3′-diaminobenzidine (DAB). We used brain sections of naive 3 × Tg-AD mice at 18 months of age as a control to examine spontaneously developed Tau pathology in this mouse model.

Free-floating brain sections were washed with PBS and permeabilized with 0.3% Triton X-100 in PBS. Endogenous peroxidase activity was abolished by treatment with 0.3% H_2_O_2_ in PBS. The sections were then blocked with normal goat serum, followed by incubation either with AT8 or with antibody PHF-1 overnight at 4 °C, followed by washing in PBS and incubation with horseradish peroxidase-conjugated goat antimouse IgG (1:2000; Jackson ImmunoResearch, West Grove, PA, USA) for 2 h at room temperature. After washing with PBS, sections were developed with 0.05% DAB plus 0.015% H_2_O_2_ in PBS. Sections were then mounted on precleaned, positively charged microscopic slides; counterstained with Mayer’s hematoxylin; dehydrated; cleared in Histo-Clear (National Diagnostics, Atlanta, GA, USA); and coverslipped. Stained sections were visualized, and photomicrographs were taken using a Nikon 90i research microscope. Staining was color-thresholded using ImageJ for Windows software (https://imagej.nih.gov/ij/), and the percentage of area occupied by AT8 or PHF-1 staining in the somatodendritic compartment of individual CA1 neurons with Tau inclusions was quantified at × 60 magnification.

### Western blot analysis

For Western blot analysis, AD brain homogenate (10%) prepared in 20 mM Tris-HCl, pH 8.0, 0.32 M sucrose, 10 mM β-mercaptoethanol, 5 mM MgSO_4_, 1 mM EDTA, 10 mM glycerophosphate, 1 mM Na_3_VO_4_, and 50 mM NaF with phosphatase and protease inhibitors was centrifuged at 15,000 × *g* for 30 minutes [18]. The extract and the AD p-Tau samples were boiled in 2× Laemmli buffer for 5 minutes.

N2a cells (the mouse neuroblastoma cell line) were harvested in the exponential growth phase and lysed in Laemmli SDS sample buffer with Roche cOmplete Mini Protease Inhibitor mixture (Roche Diagnostics, Indianapolis, IN, USA) and directly boiled for 5 minutes. Aβ_1–42_ used for Western blotting was synthesized commercially (a gift from Dr. David Miller).

Protein concentration was measured by using the Pierce^TM^ 660 nm protein assay (Thermo Fisher Scientific, Rockford, IL, USA). The samples were resolved in 10% SDS-PAGE and electrotransferred onto Immobilon-P membrane (MilliporeSigma, Burlington, MA, USA). The blots were then probed with primary antibodies PHF-1 and transactive response DNA-binding protein 43 (TDP-43) (Cell Signaling Technology) and developed with the corresponding horseradish peroxidase-conjugated secondary antibody and enhanced chemiluminescence (ECL) kit (Thermo Fisher Scientific). For Aβ, the samples were resolved in 5–20% gradient SDS-PAGE and electrotransferred onto nitrocellulose membrane. The blots were then probed with Aβ antibody 4G8 (BioLegend, San Diego, CA, USA) and developed with the corresponding horseradish peroxidase-conjugated secondary antibody and ECL kit (Thermo Fisher Scientific).

### Statistical analysis

Data were analyzed using Prism version 5.0 software (GraphPad Software Inc., La Jolla, CA, USA) and one-way or two-way analysis of variance (as appropriate) followed by a Bonferroni post hoc test. Intergroup comparisons were performed using paired or unpaired two-tailed *t* tests. All data are presented as mean ± SEM, and *P* < 0.05 was considered a statistically significant difference.

## Results

### AD p-Tau seeds Tau pathology in a time-dependent manner in 3 × Tg-AD mice

In a recent study, we showed that intrahippocampal injection of oligomeric AD p-Tau into the hippocampus of 3-month-old h-Tau-transgenic mice produced spread of Tau pathology in the brain at 11 months after injection [17]. To develop a practical mouse model in which Tau spread can be studied in a relatively short period, we employed 11- to 12-month-old 3 × Tg-AD mice. Unlike the original report of Oddo et al. [20], the 3 × Tg-AD mice bred in our colony develop amyloid plaques at around 13 months of age and Tau pathology even later. To determine the potency of the AD p-Tau to template the host Tau into NFTs in 3 × Tg-AD mice, we unilaterally injected 0.35 μg of the oligomeric Tau protein into the right hippocampus of 11- to 12-month-old female 3 × Tg-AD mice and evaluated Tau pathology at 3 and 6 weeks after the injection (Fig. 1a). Compared with AD brain extract, the AD p-Tau was highly enriched in hyperphosphorylated Tau as seen on Western blots developed with phospho-Tau antibody PHF-1 (Fig. 1b). Furthermore, the AD p-Tau preparation did not have detectable levels of either Aβ or TDP-43 (Fig. 1c, d).

**Fig. 2:**
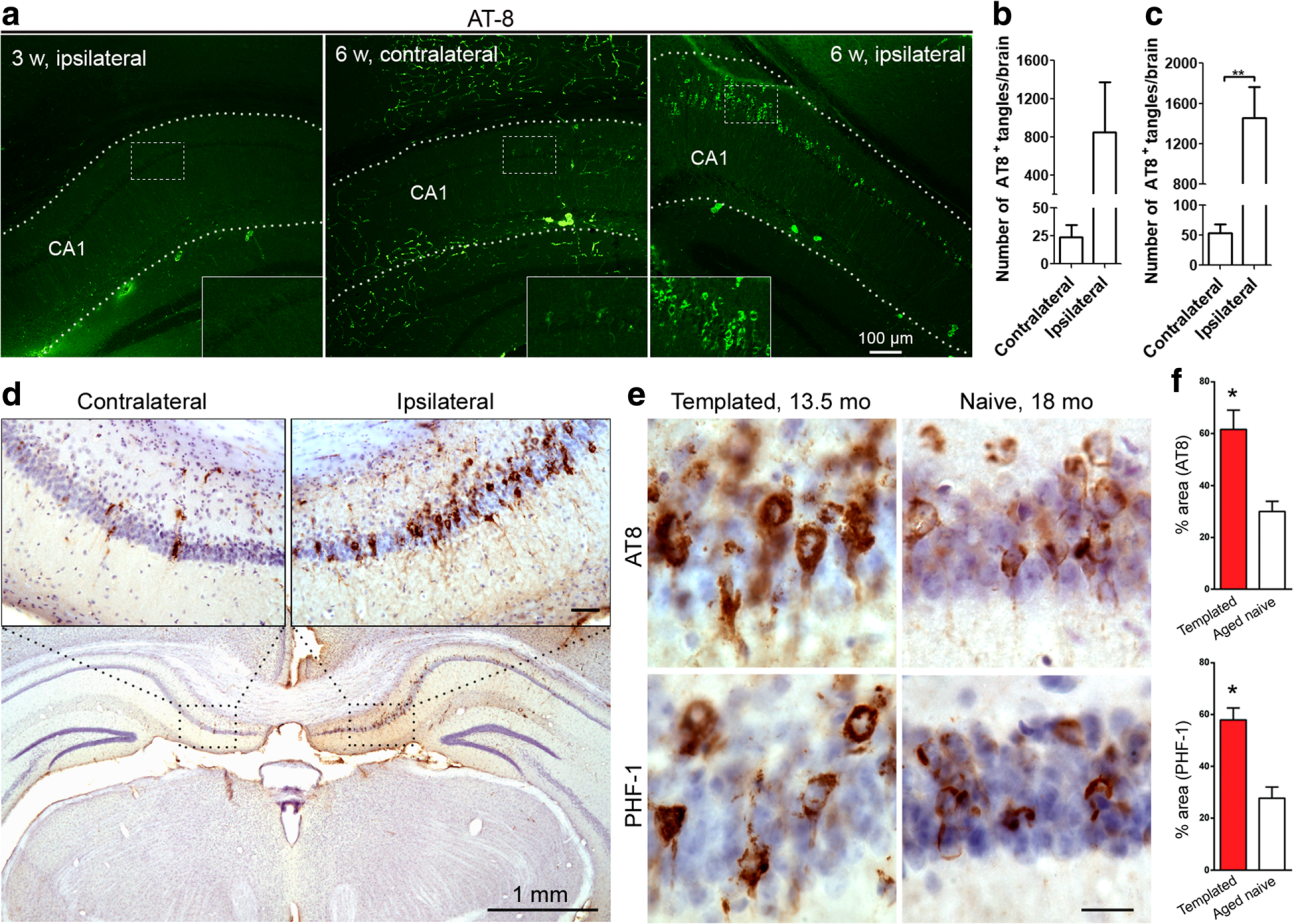
Alzheimer’s disease abnormally hyperphosphorylated Tau (AD p-Tau) templates the host Tau into neurofibrillary pathology in a time-dependent manner in triple-transgenic Alzheimer’s disease (3 × Tg-AD) mice. AD p-Tau (0.35 μg/2.5 μl saline) isolated from AD cerebral cortex was unilaterally injected into hippocampi of 11- to 12-month-old female 3 × Tg-AD mice. These animals shown in (**a**) and (**b**) were a separate group from those shown in Fig 1a and were employed solely to assess the time-dependent seeding activity and templating of the host Tau into neurofibrillary tangles in the ipsilateral hippocampus and spread of Tau pathology to the contralateral hippocampus. At 3 and 6 weeks after AD p-Tau injection, the animals were killed, and coronal sections of their brains were immunostained with AT8 (pSer^202^/pThr^205^) antibody. **a** Ipsilateral hippocampus at 3 weeks after AD p-Tau injection (*left panel*), contralateral side (*middle panel*), and ipsilateral side (*right panel*) at 6 weeks after AD p-Tau injection. CA1 area is marked by *dashed lines*. High-magnification images are shown as insets. Scale bar = 100 μm. **b** The number of AT8 intensely stained neurofibrillary tangles was counted from mice at 6 weeks after AD p-Tau injection (*n* = 2) at × 10 magnification. No tangles were observed in either the contralateral or ipsilateral side of mice at 3 weeks after AD p-Tau injection (*n* = 2). **c** The number of AT8 high-intensity neurofibrillary tangles was counted from mice at 7.5 weeks after AD p-Tau injection (AD p-Tau/immunoglobulin G [IgG]; *n* = 7) at × 10 magnification. **d** Immunostaining with AT8 for brain sections from AD p-Tau/IgG mice at 7.5 weeks after AD p-Tau injection. The contralateral hippocampus showed only a few tangles. Magnified views of boxed areas are shown. **e** Tau pathology load (AT8 and PHF-1) in CA1 neurons after AD p-Tau injection was significantly higher than that seen in a group (not shown in Fig. 1a) of 18-month-old naive 3 × Tg-AD mice (*n* = 3). **f** Bar graphs showing percentage of immune-positive area in somatodendritic compartment of CA1 pyramidal neurons at × 60 magnification. Not shown in this figure is that no primary antibody immunostaining controls were employed for both immunofluorescence and 3,3′-diaminobenzidine staining and were found to show only negative/background staining. Data are presented as mean ± SEM and analyzed by unpaired two-tailed Student’s *t* tests (*n* = 3 animals/group). *P < 0.05 compared with naive female 18-month-old 3 × Tg-AD mice. Scale bars = 1 mm for low magnification and 50 μm for higher magnification in (**d**); and scale bar = 20 μm in (**e**)

At 3 weeks after AD p-Tau injection, we found low-intensity AT8 staining, and at 6 weeks we found high-intensity AT8 staining, in the ipsilateral hippocampus, but only low-intensity AT8 staining at these two time points in the contralateral hippocampus (Fig. 2a). The number of the intensely stained neurons was several-fold higher in the ipsilateral than in the contralateral hippocampus (Fig. 2b). These results indicate that AD p-Tau has seeding activity and can template host Tau into NFTs as early as 6 weeks after AD p-Tau injection in 11- to 12-month-old 3 × Tg-AD mice. This process is faster than what we previously found in h-Tau mice, in which Tau seeded and NFTs were observed 9 months after injections [17].

### AD p-Tau successfully seeds and templates host Tau into neurofibrillary tangles in 3 × Tg-AD mice

Female 3 × Tg-AD mice develop amyloid plaques starting at ~ 9–13 months and NFTs starting ~ 12–15 months of age, and the pathologies are restricted predominantly to the hippocampus, amygdala, and cerebral cortex [20–22]. Thioflavin-S staining that recognizes β-pleated sheets is commonly used to label amyloid plaques and NFTs [28, 29]. On the basis of our findings described above that showed overt Tau pathology 6 weeks after injection of AD p-Tau, we perfused the remaining study mice, which by then were 7.5 weeks post-AD p-Tau injection, to evaluate the seeding and spread of Tau pathology.

Immunohistochemical staining showed that AD p-Tau seeded robust NFT-like pathology in the ipsilateral hippocampus of 11- to 12-month-old 3 × Tg-AD mice at 7.5 weeks after unilateral AD p-Tau injection (Fig. 2c–f). The number of AT8 high-intensity positive tangles in the AD p-Tau-injected hippocampus were 0, 800, and 1500 at 3 (AD p-Tau/saline; *n* = 2), 6 (AD p-Tau/saline; *n* = 2), and 7.5 (AD p-Tau/IgG; *n* = 7) weeks after AD p-Tau injection, which clearly demonstrated that AD p-Tau time-dependently templated host Tau into tangles in 3 × Tg-AD mice (Fig. 2b, c). AT8 immnunostained neurons were located mainly in CA1 and subiculum subfields of the hippocampus, whereas CA3 and dentate gyrus were spared from Tau pathology (Fig. 2d). In the contralateral hippocampus, however, fewer neurons were positive for AT8 or PHF-1 (Fig. 2d). Intriguingly, neurons with templated Tau pathology in 13.5-month-old 3 × Tg-AD mice showed even much higher, approximately twofold, load of tau inclusions compared with the spontaneously developed Tau pathology in 18-month-old naive female 3 × Tg-AD mice (Fig. 2e and f).

**Fig. 3:**
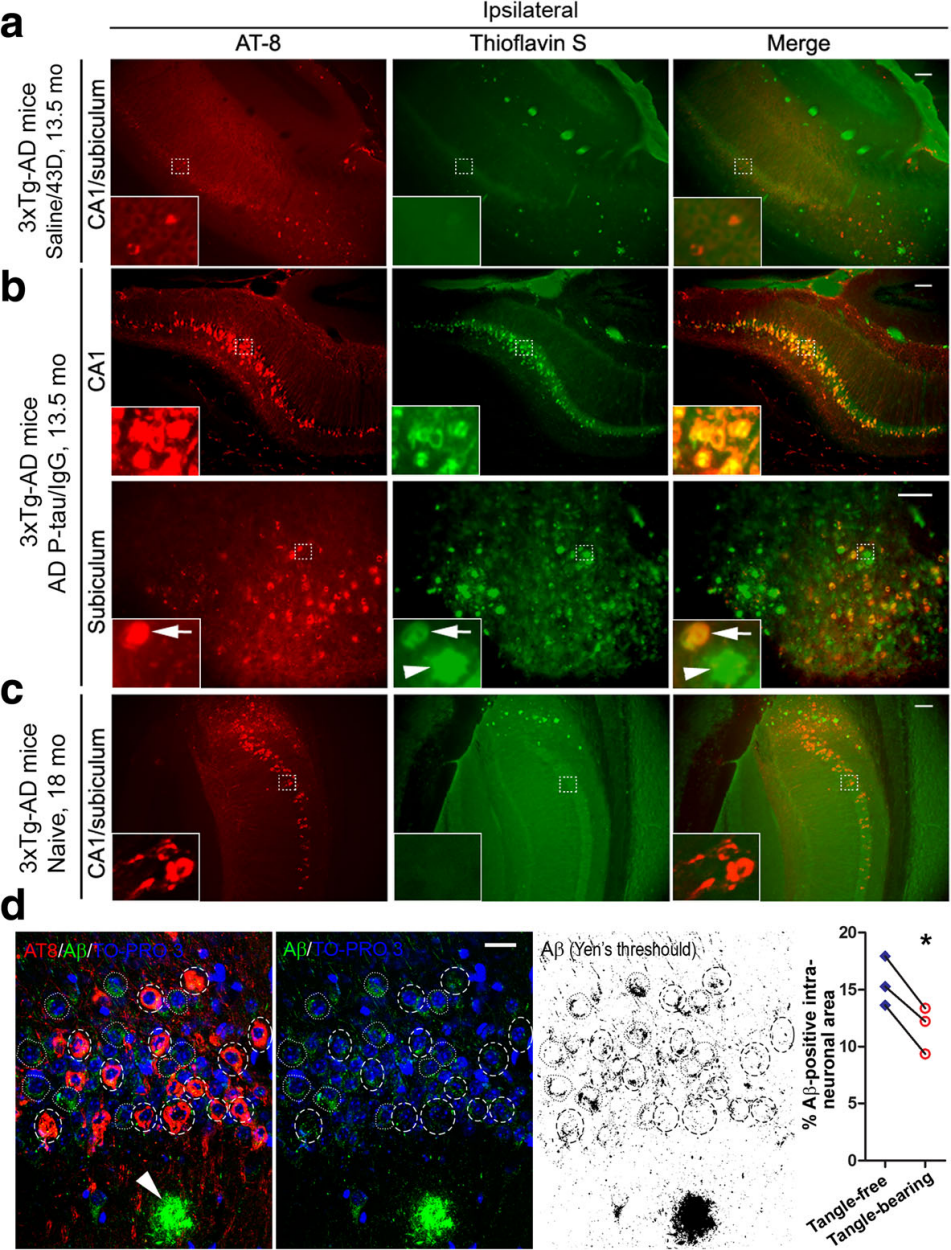
Alzheimer’s disease abnormally hyperphosphorylated Tau (AD p-Tau) seeds and templates the host Tau into neurofibrillary pathology at 7.5 weeks after AD p-Tau injection in triple-transgenic Alzheimer’s disease (3 × Tg-AD) mice. AD p-Tau (0.35 μg/2.5 μl saline), or saline (2.5 μl) as vehicle control, was unilaterally injected into the right hippocampus of 11- to 12-month-old female 3 × Tg-AD mice, and the mice were perfused at 7.5 weeks after injection. **a** AT8 and thioflavin-S staining in the 3 × Tg-AD mice injected with saline and treated with 43D. **b** AT8 and thioflavin-S staining in CA1 and subiculum in the 3 × Tg-AD mice injected with AD p-Tau and treated with mouse immunoglobulin G (IgG). **c** AT8 and thioflavin-S staining in naive 18-month-old female 3 × Tg-AD mice. **d** Double-immunofluorescence staining with AT8 and anti-amyloid-β (anti-Aβ) (D54D2) in brain sections of mice injected with AD p-Tau and treated with control mouse IgG. Tangle-bearing neurons (*dashed circles*) showed neither Aβ-positive filamentous inclusions nor increased Aβ somatodendritic immunoreactivity as compared with tangle-free neurons (*dotted circles*). Aβ staining was thresholded using Yen’s arithmetic and quantified using ImageJ software, and results were expressed as average percentage area occupied by Aβ staining in the somatodendritic compartment of tangle-bearing neurons compared with adjacent tangle-free neurons at × 60 magnification (*n* = 3 mice). High-magnification views of the boxed regions are shown as insets. *Arrows* = tangles; *arrowheads* = Aβ plaques. *P < 0.05, paired *t* test. Scale bars = 100 μm (**a**–**c**) and 20 μm (**d**)

We employed double labeling with immunofluorescence and thioflavin-S staining to further characterize the templating of Tau pathology by AD p-Tau in 3 × Tg-AD mice. In the ipsilateral hippocampus of saline-injected mice, we found amyloid plaques with thioflavin-S-positive staining, but very few neurons showed high-intensity AT8 staining (Fig. 3a). Additionally, the few neurons that showed high-intensity AT8 staining were found negative with thioflavin-S staining (Fig. 3a). Importantly, the mice injected with AD p-Tau showed an increased density of neurons with high-intensity AT8 staining, and these neurons were also thioflavin-S-positive in the CA1 and subiculum areas (Fig. 3b). In contrast, the naive 18-month-old 3 × Tg-AD mice showed intense AT8 staining similar to that of the AD p-Tau-injected 13.5-month-old mice, but they were thioflavin-S-negative (Fig. 3c). To further characterize the identity of the filamentous intraneuronal inclusions templated by AD p-Tau preparation, we coimmunostained brain sections with AT8 and rabbit monoclonal antibody against Aβ, which recognizes both Aβ_40_ and Aβ_42_. We found that AT8-positive tangles were negative for Aβ (Fig. 3d). In addition, tangle-bearing neurons showed no increase in Aβ immunoreactivity in the somatodendritic compartment, as compared with tangle-free neurons (Fig. 3d). These results indicate that AD p-Tau can rapidly seed and template host tau into NFTs in the 11- to 12-month-old 3 × Tg-AD mice.

### Immunization with 43D blocks AD p-Tau seeding and Tau pathology propagation in 3 × Tg-AD mice

**Fig. 4:**
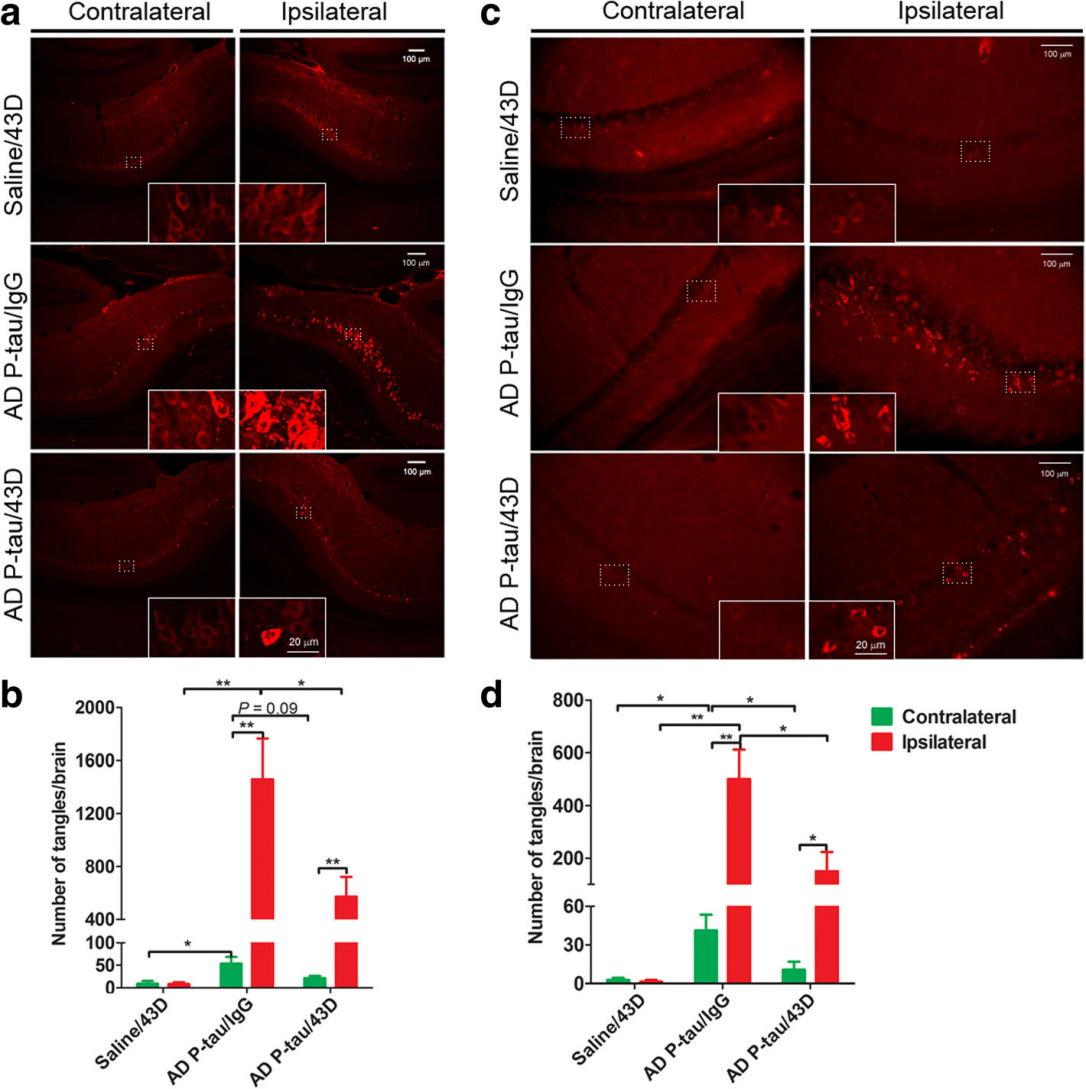
Immunization with 43D monoclonal antibody blocks Alzheimer’s disease abnormally hyperphosphorylated Tau (AD p-Tau) seeding and inhibits the spread of neurofibrillary pathology in triple-transgenic Alzheimer’s disease (3 × Tg-AD) mice. AD p-Tau (0.35 μg in 2 μl of saline) isolated from AD cerebral cortex was unilaterally injected into the right hippocampus of 11- to 12-month-old female 3 × Tg-AD mice or saline as a vehicle control. The mice were perfused at 7.5 weeks after AD p-Tau injection, and coronal sections were immunostained with monoclonal antibodies AT8 and PHF-1. **a** Representative image of AT8 staining of the contralateral and ipsilateral CA1 areas of the brain. High-magnification images are shown as insets. **b** The number of tangles (AT8-positive somatodendritic profiles) was counted from 22 to 25 serial coronal sections per mouse (every fourth section of the brain) at × 10 magnification. **c** Representative image of PHF-1 staining in the contralateral and ipsilateral CA1 areas. High-magnification images are shown as insets. **d** The number of tangles (PHF-1-positive somatodendritic profiles) were counted from 22 to 25 serial coronal sections per mouse (every fourth section of the brain) at × 10 magnification. Saline/43D (*n* = 6), AD p-Tau/43D (*n* = 6), AD p-Tau/IgG (*n* = 7). *P < 0.05, **P < 0.01

Our previous studies showed that immunization with mouse monoclonal antibody 43D targeting the N-terminal projection domain of Tau decreased Tau pathology and improved cognitive deficits in moderate to severe stages of Tau pathology in 3 × Tg-AD mice [18, 19]. To investigate whether 43D monoclonal antibody can block AD p-Tau seeding and inhibit the spread of templated host Tau inclusions in 3 × Tg-AD mice, we immunized mice with six weekly intravenous doses of 43D or as a control with mouse IgG. On the day of the second dose of immunization, AD p-Tau (0.35 μg/2.5 μl) was unilaterally injected into the right hippocampus (Fig. 1a). The mice were killed at 7.5 weeks after AD p-Tau injection, and serial coronal sections were collected. No high-intensity AT8 staining was observed in either the ipsilateral or contralateral hippocampus of the mice with saline injection into the hippocampus and treated with 43D (Fig. 4a). However, many neurons in the ipsilateral hippocampus showed prominent Tau inclusions that were labeled with high-intensity AT8 staining in the mice with AD p-Tau injection and treated with mouse IgG (Fig. 4a), which indicates that injected AD p-Tau acts as Tau seeds and induces the host-overexpressed Tau to form NFTs in seeded neurons. Importantly, immunization with 43D dramatically decreased Tau pathology in the ipsilateral hippocampus of the mice injected with AD p-Tau compared with mouse IgG-treated controls. We counted the double AT8 and thioflavin-S-positive neurons from serial 22–25 coronal sections per mouse, which were every fourth section of the brain. The quantification data showed that immunization with 43D significantly decreased Tau pathology in the ipsilateral hippocampus (Fig. 4b).

To determine whether pathological Tau induced by AD p-Tau can spread to the different brain regions distant from the injection sites, we analyzed and compared Tau pathology between the ipsilateral and contralateral hippocampus. We found only a few AT8 intensely stained neurons in the mice injected with saline and treated with 43D, and there was no difference between ipsilateral and contralateral hippocampi (Fig. 4a and b). However, many AT8 intensely positive NFTs were found in the contralateral hippocampus of the mice injected with AD p-Tau and treated with mouse IgG. The number of AT8 intensely stained neurons in the mice injected with AD p-Tau was several-fold higher than that in the mice injected with saline in both the ipsilateral and contralateral hippocampus, suggesting that AD p-Tau was taken up by neurons at the injection site and that the Tau pathology from the seeded neurons of the ipsilateral hippocampus spread to the contralateral hippocampus (Fig. 4a and b). Compared with mouse IgG treatment, immunization with monoclonal antibody 43D showed a clear trend (*P* = 0.09) to decrease the number of AT8 intensely stained neurons in the contralateral hippocampus, though it did not reach statistical significance (Fig. 4a and b). Importantly, similar results were also found with PHF-1 staining (Fig. 4c and d). These findings indicate that immunization with 43D blocks the AD p-Tau induced seeding and inhibits the spread of Tau.

### AD p-Tau accelerates amyloid plaque pathology

**Fig 5:**
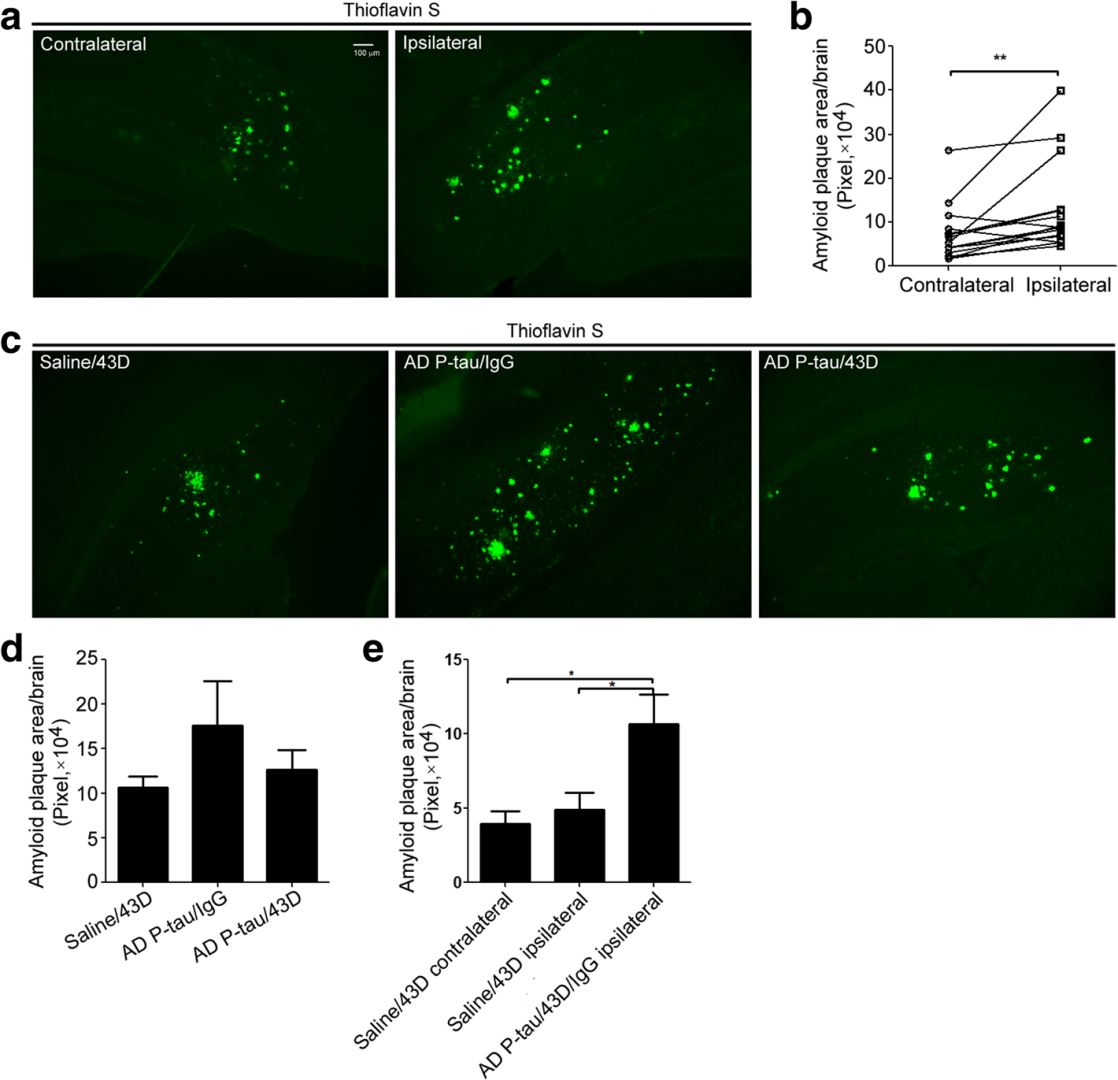
Alzheimer’s disease abnormally hyperphosphorylated Tau (AD p-Tau) injection accelerates the development of amyloid-β (Aβ) plaques in triple-transgenic Alzheimer’s disease (3 × Tg-AD) mice. AD p-Tau (0.35 μg in 2 μl of saline) isolated from autopsied AD cerebral cortex was unilaterally injected into the right hippocampus of 11- to 12-month-old female 3 × Tg-AD mice or saline as a vehicle control. The mice were perfused at 7.5 weeks after AD p-Tau injection, and the coronal sections were stained with thioflavin-S. **a** Representative images of thioflavin-S staining of plaques in the contralateral and ipsilateral subiculum of mice in the AD p-Tau/immunoglobulin G (IgG) group. **b** Plaque areas in the contralateral and ipsilateral sides of the brain were calculated at × 10 magnification from 22 to 25 serial coronal sections per mouse in AD p-Tau/43D (*n* = 6) and AD p-Tau/IgG (*n* =7) groups. **P < 0.01, paired *t* test. **c** Representative images of thioflavin-S staining of plaques in the ipsilateral subiculum of the mice in saline/43D, AD p-Tau/IgG, and AD p-Tau/IgG groups. **d** Plaque areas were calculated at × 10 magnification from 22 to 25 serial coronal sections per mouse (every fourth section of the brain) in mice treated with saline/43D (*n* = 6), AD p-Tau/IgG (*n* = 7), and AD p-Tau/43D (*n* = 6). **e** Plaque areas in the contralateral and ipsilateral hippocampus in saline/43D mice (*n* = 6) and in the ipsilateral hippocampus in AD p-Tau/43D or IgG (*n* = 13) were measured at × 10 magnification from 22 to 25 serial coronal sections per mouse (every fourth section of the brain). A two-tailed *t* test was used for ipsilateral side between saline/43D and AD p-Tau/43D and AD p-Tau/IgG mice. A paired *t* test was used between contralateral and ipsilateral hippocampus of saline/43D mice. *P < 0.05

Previously, we discovered that immunization with Tau antibody 43D can inhibit not only Tau but also Aβ pathology in 3 × Tg-AD mice [18, 19]. To investigate whether AD p-Tau injection can promote the formation of amyloid plaques, we compared the amyloid plaque load with thioflavin-S staining between the ipsilateral and contralateral brain in AD p-Tau/IgG (*n* = 7) and AD p-Tau/43D (*n* = 6) mice. We found a higher amyloid plaque burden in the AD p-Tau-injected side of the brain than in the contralateral brain, but we found a similar level of amyloid plaque load between the saline-injected side and the contralateral side (Fig. 5a, b, e). Importantly, the AD p-Tau-injected side had more amyloid plaque load than the saline-injected side (Fig. 5e). These data indicate that AD p-Tau can accelerate the development of Aβ pathology. Consistently, the amyloid plaque load was larger in the AD p-Tau-injected animals than in saline/43D and AD p-Tau/43D mice; 43D treatment showed a trend to decrease the plaque load in the AD p-Tau/43D mice compared with AD p-Tau/IgG mice (Fig. 5c and d).

## Discussion

We recently discovered that immunotherapy targeting the N-terminal projection domain of Tau with mouse monoclonal antibody 43D (Tau 6–18) decreases both Tau and Aβ pathologies and rescues memory impairment in 3 × Tg-AD mice [18, 19]. However, whether immunization with 43D can block AD p-Tau seeding and prevent the spread of Tau pathology in vivo was not known. The present study shows, for the first time to our knowledge, that immunization with 43D antibody can block AD p-Tau seeding and the consequent spread of neurofibrillary pathology in 3 × Tg-AD mice.

It has been well established that 0.1–1% of circulating antibodies can enter the brain [30, 31]. Mouse IgG has a half-life of about 1 week in plasma [32, 33]. Researchers in several studies have reported that passive immunization with high doses of antibodies (15–30 mg/kg, i.p.) decreased Tau pathology and rescued memory dysfunction [15, 34, 35]; however, Lee et al. [36] did not find a dose-dependent decrease of Tau pathology in Tau P301L mice treated once weekly with intraperitoneal injections of anti-pS409-Tau antibody at 3, 10, or 30 mg/kg for 3 months. In our studies, a dose of 43D antibody (100 μg/mouse ~ 3.5 mg/kg, i.p., once weekly for 4 weeks, or 15 μg/mouse ~ 0.5 mg/kg, i.v., once weekly for 6 weeks) decreased Tau pathology and rescued memory deficits [18, 19]. In the present study, we found that Tau antibody 43D at an i.v. dose of ~ 0.5 mg/kg blocks AD p-Tau-induced seeding and spread of Tau pathology in 3 × Tg-AD mice. Consistent with our studies, passive immunization targeting Tau with a dose similar to the one we used was shown to improve memory in AD mouse models [37, 38]. A single intravenous injection of 30 μg of oligomeric Tau antibody was reported to reverse both locomotor and memory deficits in a mouse model of tauopathy [39].

In our previous work, we found that AD p-Tau templates host Tau into neurofibrillary pathology, which shows as both high-intensity AT8- and thioflavin-S-positive staining in h-Tau mice [17]. Therefore, in the present study, we employed positive high-intensity AT8 staining to determine the seeding and spread of Tau pathology in 3 × Tg-AD mice. We found only weak AT8 staining in both the contralateral and ipsilateral hippocampus in saline-injected 13.5-month-old 3 × Tg-AD mice. However, high-intensity AT8 staining that colocalized with thioflavin-S staining was apparent at 6–7.5 weeks after AD p-Tau injection in 3 × Tg-AD mice. Interestingly, although we observed similar high-intensity AT8 staining in naive 18-month-old female 3 × Tg-AD mice, these AT8-positive neurons were mostly thioflavin-S-negative. One possible explanation is that high-intensity AT8 staining, even in 18-month-old 3 × Tg-AD mice, represents a nonfibrillized hyperphosphorylated Tau (i.e., a less advanced stage than NFTs). Thus, it appears that Tau pathology induced by AD p-Tau in 3 × Tg-AD mice is similar to that seen in AD. The prion-like seeding and propagation of Tau pathology in the present study is consistent with previous studies in which investigators employed intracerebral injection of brain homogenates from Tau-transgenic mice [9, 40, 41] and humans with pathologically confirmed AD or related tauopathies [10, 42], or synthetic tau fibrils formed in vitro [11, 43], into h-Tau-transgenic mice or wild-type mice [10, 44] to show the conversion of the host Tau into insoluble NFT-like aggregates and their spread in recipient mice. The spread of Tau pathology in a progressive manner to the hippocampus was also shown in human P301L Tau-transgenic mice that express the transgene apparently specifically in layer II neurons of the EC [12, 13].

Previous studies showed that seeded Tau pathology can spread to anatomically connected areas via synaptic connection [12, 13, 40, 41, 43]. In a recent study, we found seeded somatodendritic Tau pathology in axonally connected upstream cortex areas, suggesting that hyperphosphorylated Tau seeds isolated from AD brain might have templated or been transported retrogradely to the somatodendritic compartment [17]. Because the left and right hippocampi innervate each other, we postulate that axonal connection could be the main approach through which Tau is spread to the contralateral side. However, the possibility of Tau to spread to the contralateral hippocampus through interstitial/ventricular fluid cannot be ruled out.

We previously found the spread of Tau pathology 11 months after AD p-Tau injection in h-Tau mice [17]. In the present study, we found the seeding and spread of Tau pathology induced by AD p-Tau as early as 6 weeks after AD p-Tau injection in a time-dependent manner similar to that observed in human tauopathies and its inhibition with Tau antibody 43D in 3 × Tg-AD mice. These features make the AD p-Tau-injected 3 × Tg-AD mice a practical in vivo model to screen Tau-based treatment approaches.

Recent studies have shown that Tau can be released from intact neurons into the extracellular space as a physiological process that is independent from neuronal death [45–47], which indicates that constitutive Tau release into extracellular space could play an important role in the spread of Tau pathology in AD and related tauopathies. Importantly, mostly N-terminal, but not C-terminal, fragments are found in extracellular Tau in AD cerebrospinal fluid (CSF) [48, 49]. Extracellular Tau seeds can enter neurons and further induce intracellular Tau accumulation and subsequent spreading of Tau pathology. Therefore, extracellular Tau plays a pivotal role in the pathogenesis of AD and related tauopathies and provides a potential target for the treatment of AD and related tauopathies. In the present study, we did not collect and analyze CSF or interstitial levels of Tau and thus do not know whether the inhibition of Tau pathology observed by immunization with 43D was due to clearance of only extracellular or both intracellular and extracellular Tau; only a fraction of the antibody is known to be taken up by neurons [50, 51]. Tau antibodies can enter neurons through clathrin-mediated endocytosis following binding to low-affinity FcγII or FcγIII receptors on neurons [50, 51] or through other mechanisms [34, 52], and they interact with intracellular Tau that inhibits its aggregation and prevents its release into the extracellular space and subsequent spread in the brain [53, 54]. Thus, intracellular interaction between 43D antibody and Tau may also contribute to the inhibition of the seeding and spread of Tau pathology.

According to the Aβ cascade hypothesis, Tau pathology is believed to be downstream of Aβ pathology [55]. However, studies of human AD and control brains of various ages revealed that Tau pathology can also precede Aβ pathology in aged and AD brains [56, 57]. In the present study, we found that AD p-Tau injection enhanced the Aβ plaque load in the ipsilateral side compared with the contralateral side of the brain, and immunization with 43D shows a trend to reduce Aβ pathology. Previously, we found that immunization with 43D can decrease Aβ pathology in mice [18, 19], which was confirmed recently by Sigurdsson’s group, who showed that active immunization with a Tau peptide reduces both Tau and Aβ pathologies in 3 × Tg-AD mice [58]. These findings indicate that Tau pathology may affect Aβ pathology. The possible mechanism by which Tau pathology affects Aβ pathology is currently not understood. Although we did not detect any Aβ in the AD p-Tau preparation, the presence of undetectable amounts of Aβ oligomers sufficient to induce Aβ pathology cannot be ruled out. Alternatively, Tau pathology could possibly enhance Aβ pathology by promoting neurodegeneration-associated amyloidogenic processing of APP and/or by inhibiting the degradation of Aβ. Similarly, inhibition of Tau pathology by immunization with Tau antibody 43D could have increased neuronal plasticity and consequently reduced Aβ pathology by reducing the amyloidogenic processing of APP. In our previous study, we found that immunization with 43D increased the levels of C1q, the first protein in the complement cascade, and C9, the late-stage activation marker, and promoted microglial activation and aggregation around Aβ plaques [18]. The complement system plays primary role in innate and adaptive immune response, and several studies indicate that complement may also play an important role in the pathogenesis and progression of AD [59]. C1q can protect cultured primary neurons against Aβ-induced neurotoxicity [60]. Knockout of C3 or overexpression of complement receptor-related protein y (Crry), an inhibitor of complement C3 convertase, increases the amyloid load but reduces phagocytic microglia [61, 62]. Microglia are the phagocytes of the brain and express complement receptor CR3. C3 promotes plaque clearance via triggering of Aβ phagocytosis by microglia [63, 64]. Therefore, the activation of the complement system by passive immunization with monoclonal antibody 43D might be involved in eliminating aggregated and toxic proteins.

## Conclusions

The present study shows that the 3 × Tg-AD mouse intracerebrally injected with AD p-Tau provides a practical model of seeding and spread of tau pathology and acceleration of Aβ pathology. Tau pathology can promote Aβ pathology. Immunization with 43D blocks the AD p-Tau-induced seeding and the spread of Tau pathology and acceleration of Aβ pathology. These studies indicate that immunization with 43D against N-terminal projection domain of Tau (Tau 6–18) represents a potential treatment opportunity for AD and related tauopathies.

## Abbreviations

Aβ: Amyloid-β
AD: Alzheimer’s disease
AD p-Tau: AD abnormally hyperphosphorylated Tau
CA: Cornu amonis
CSF: Cerebrospinal fluid
DAB: 3,3′-Diaminobenzidine
EC: Entorhinal cortex
ECL: Enhanced chemiluminescence
EDTA: Ethylenediaminetetraacetic acid
h-Tau: Human Tau
IgG: Immunoglobulin G
NFT: Neurofibrillary tangle
TDP-43: Transactive response DNA-binding protein 43
3 × Tg-AD: Triple-transgenic Alzheimer’s disease mouse model
